# BMI mediates the association of serum uric acid with bone health: a cross-sectional study of the National Health and Nutrition Examination Survey (NHANES)

**DOI:** 10.1186/s12891-024-07595-8

**Published:** 2024-06-19

**Authors:** Jiayuan Tu, Xiaoqiao Mo, Xiangda Zhang, Zihao Chen, Lijuan Xi, Chunhui Wu, Xiangchan Zeng, Tian Xie

**Affiliations:** 1 School of nursing and school of public health, Yangzhou University, Yangzhou, Jiangsu 225000 China; 2 Department of Operating Room, Xinhua Hospital, Shanghai Jiao Tong University School of Medicine, Shanghai, 200000 China; 3 Department of Public Health and Preventive Medicine, Monash University, Melbourne, VIC 276199 Australia; 4 College of Physical education, Yangzhou University, Yangzhou, Jiangsu 225000 China; 5 Department of Cardiology, Zhongda Hospital Southeast University, Nanjing, Jiangsu 210000 China; 6 Gynacology Department, Shenzhen Nanshan Medical Group Headquarter, Shenzhen, Guangdong 518000 China; 7 Department of General Surgery, Shanghai Ninth People’s Hospital, Shanghai Jiao Tong University School of Medicine, Shanghai, 200000 China

**Keywords:** Uric acid, Bone mineral density, NHANES, Osteoporosis, Osteopenia

## Abstract

**Background:**

The associations between serum uric acid and osteoporosis or osteopenia remain controversial, and few studies have explored whether BMI acts as a mediators in the association between the SUA and OP/ osteopenia.

**Objective:**

To explore the relationship between serum uric acid and osteoporosis or osteopenia among US adults.

**Methods:**

A cross-sectional study was conducted to examine the association between serum uric acid and osteoporosis or osteopenia from four cycles of NHANES. Binary logistic regression models and restricted cubic spline models were used to evaluate the association between serum uric acid and osteoporosis or osteopenia, and interaction analysis was used to test the differences between subgroups. Mediation analysis was utilized to investigate whether BMI acts as a mediator in the association between SUA and OP/ osteopenia.

**Results:**

12581 participants aged ≥ 18 years were included. A U-shape nonlinear relationship between SUA and osteoporosis or osteopenia in all people was found (*P* < 0.0001, *P* for nonlinear = 0.0287). There were significant interactions in age subgroups (*P* for interaction = 0.044), sex subgroups (*P* for interaction = 0.005), and BMI subgroups (*P* for interaction = 0.017). We further assessed the subgroups and found the optimal range of serum uric acid levels with a lower risk of osteoporosis or osteopenia was 357–535 µmol/L in males, 327–417 µmol/L in people aged ≥ 50 years, above 309 µmol/L in people aged < 50 years, 344–445 µmol/L in people with BMI ≥ 30, and above 308 µmol/L in people with BMI < 30. BMI fully mediated the association of SUA and OP/osteopenia, with a value of -0.0024(-0.0026–-0.0021). These results were robust in sensitivity analyses.

**Conclusions:**

A complicated relationship between SUA and bone health in different populations was observed. Maintaining SUA within a specific range may be beneficial to bone health. In addition, BMI may play an important role in the association between SUA and bone health, but considering the limitations of this study, further prospective research is required.

**Supplementary Information:**

The online version contains supplementary material available at 10.1186/s12891-024-07595-8.

## Introduction

Osteoporosis (OP) or osteopenia is a complex, multifactorial, prevalent bone disorder worldwide, characterized by low bone mass and impaired microarchitectural structure. The decreased bone strength with increased fragility predisposes bones to fracture and thereby results in tremendous suffering, considerable economic costs, and higher short-term mortality risk [1–3]. Currently, it has been estimated that more than 200 million people are suffering from osteoporosis, and the number will increase dramatically in the coming decades. The International Osteoporosis Foundation estimated that one in three women over 50 years old and one in five men will experience an osteoporotic fracture [4]. Thus, potential factors for bone mineral density (BMD) need to be identified which are vital for devising public health strategies.

Serum uric acid (SUA) is an end product of purine nucleosides and free bases degradation in humans and higher primates, and it has been considered an essential endogenous potent antioxidant [5–7]. Recently, some studies have demonstrated that higher SUA might be beneficial for bone metabolism through its antioxidant properties [8, 9]. However, the other studies revealed no causal association between SUA and BMD [10, 11]. Existing studies provided somewhat conflicting results about the association between SUA and bone health, and most of the existing studies have quantified the relationship between SUA levels and BMD with simple or transformed linear models [8–12]. Accordingly, the purpose of this study was to explore the association between SUA and bone health in people aged 18 or older by using mediation analysis and a nonlinear model, restricted cubic splines (RCS), a nonlinear model based on a piecewise cubic polynomial function. These representative sample populations were derived from the NHANES (National Health and Nutrition Examination Surveys).

## Methods

### Ethical statement

This was a retrospective cross-sectional study using data from four cycles of NHANES (2007–2008, 2009–2010, 2013–2014, 2017–2018). The study was approved by the National Center for Health Statistics Research Ethics Review Board, and written consent was obtained from each participant. Data from NHANES are publicly available and anonymous, so this study received an Institutional Review Board exemption from the University of California, Los Angeles.

### Study population

**Fig. 1:**
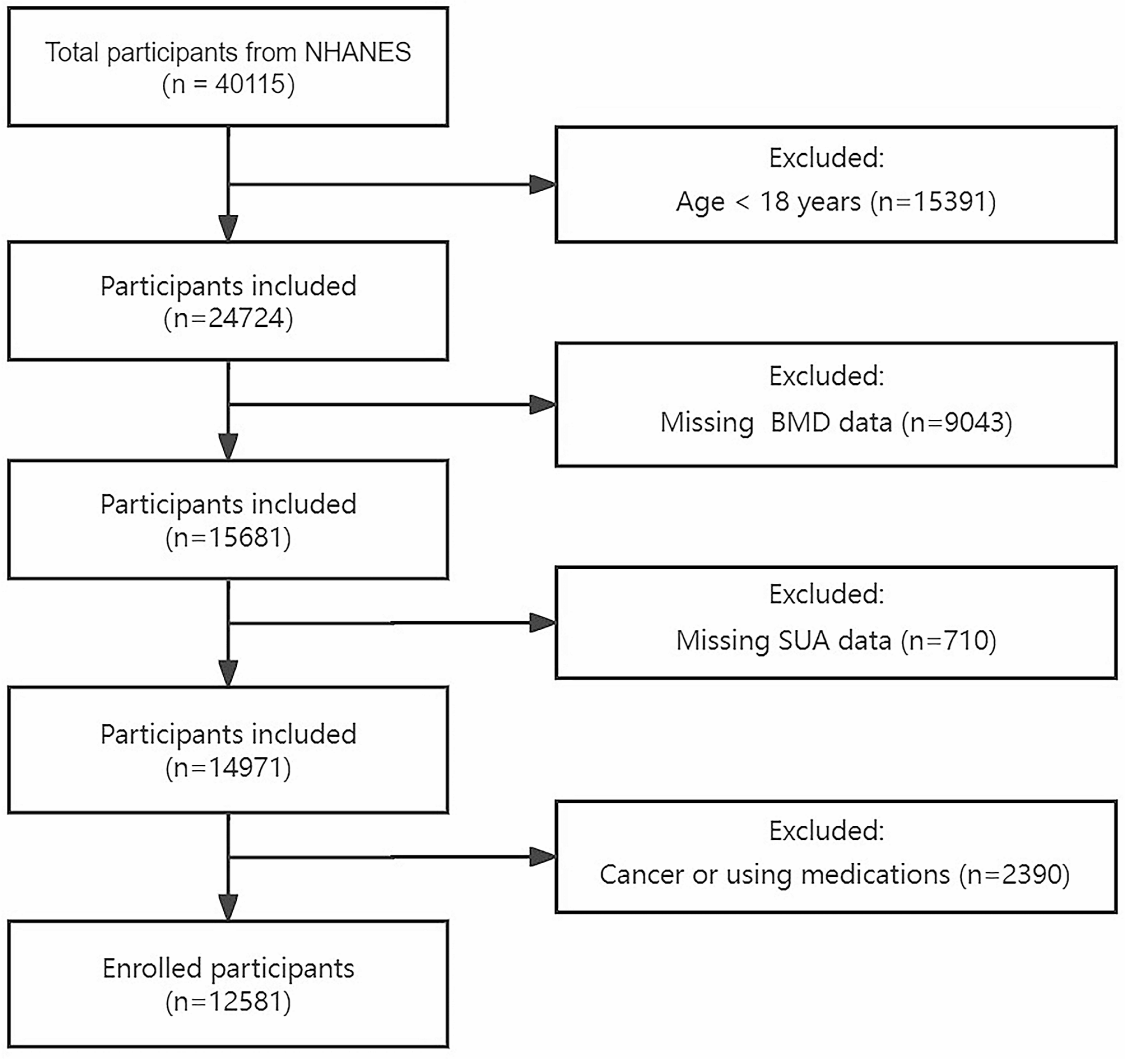
Selection of study participants in the National Health and Nutrition Examination Survey (NHANES, 2007–2010, 2013–2014, and 2017–2018)

The National Health and Nutrition Examination Survey (NHANES) is a cross-sectional representative survey that provides multitudinous information about the nutrition and health of adults and children across the United States using a complex, stratified, multistage, probability sampling design [13]. Four NHANES survey cycles (2007–2008, 2009–2010, 2013–2014, and 2017–2018) were analyzed in this study, which provided information on SUA and BMD. A total of 40,115 individuals who completed the interviews and physical examinations and responded to all relevant questions were included. 27,534 participants were excluded for the following reasons: aged < 18 years old (*n* = 15,391), missing BMD data (*n* = 9043), missing SUA data (*n* = 710), participants with cancer (*n* = 1625), or patients using medications that might affect the bone metabolism or uric acid level (such as diphosphonate, glucocorticoids, estrogen, allopurinol, benzbromarone, etc.) (*n* = 765). A total of 12,581 participants were included in the final analysis. The detailed screening process is shown in Fig. 1.

### Study variables

The exposure variable of this study was SUA, which has been measured using Beckman Synchron LX20 since 2002. The outcome variables were femoral neck and total femur BMD, measured by Dual-energy X-ray absorptiometry (DXA)with a Hologic QDR-4500 A fan-beam densitometer (Hologic, Inc., Bedford, Massachusetts), and the results were expressed as grams of bone mineral per square centimeter (g/cm^2^). DXA scans were performed by NHANES well-trained and certified radiology technologists in the NHANES mobile examination center (MEC). The mean femoral BMD of non-Hispanic white adults (20–29 years old) in the NHANES III database was defined as the reference value according to the study of Looker et al. [14]. According to the criteria recommendation of the World Health Organization, osteopenia was defined as -2.5 < T-score <-1.0 standard deviation (SD), and osteoporosis was diagnosed by T-score <-2.5 SD [15]. In the present study, OP/osteopenia was defined as meeting one of the criteria for osteopenia or osteoporosis.

In addition, the following covariates were included: sex, age, race/Hispanic origin, body mass index (BMI), high-density lipoprotein (HDL), alanine aminotransferase (ALT), asparate aminotransferase (AST), albumin (g/L), globulin(g/L), bilirubin (umol/L), alkaline phosphatase (U/L), serum calcium (mmol/L), serum 25(OH.)D (nmol/L), phosphorus (mmol/L), eGFR, total cholesterol (mmol/L), diabetes, hypertension, vigorous work activity, self-reported disease: gout, liver disease, kidney disease, history of fracture, thyroid disease, removing both ovaries. Participants who meet one of the following four criteria will be defined as having diabetes: (1) fasting blood glucose ≥ 126 mg/dl; (2) two-hour oral glucose tolerance test (OGTT) ≥ 200 mg/dl; (3) glycohemoglobin ≥ 6.5%; (4) having been told by a doctor to have diabetes. Participants who meet one of the following three criteria will be defined as having hypertension: (1) systolic blood pressure (SBP) ≥ 140 mmHg; (2) diastolic blood pressure (DBP) ≥ 90 mmHg; (3) having been told by a doctor to have hypertension. The Modification of Diet in Renal Disease (MDRD) equation was used to calculate eGFR: 186 × Scr^− 1.154^ × Age^− 0.203^ × (0.742 if female) [16].

### Statistical analysis

According to NHANES analytic guidelines, complex sampling design and sampling weights were considered in our analyses [17]. The sampling weight was calculated using the following formula: fasting subsample 8-year mobile examination center (MEC) weight = fasting subsample 2-year MEC weight/4. The characteristics of participants are described as means (SD) for continuous variables and frequencies (percentages) for categorical variables. The *t*-test and chi-square test were used to compare continuous and categorical data, respectively.

A restricted cubic spline was used to explore the association of SUA levels and OP/osteopenia prevalence flexibly. In the restricted cubic spline model, all covariates above were adjusted. The non-linearity assumption was tested by using a likelihood ratio test.

We further stratified the analyses by sex (male, female), age (< 50, ≥ 50) [17], race (Mexican American, Other Hispanic, Non-Hispanic White, Non-Hispanic Black, Other Race), BMI (< 30, ≥ 30), diabetes (no, yes), hypertension (no, yes), vigorous work activity (no, yes), gout (no, yes), liver disease (no, yes), kidney disease (no, yes), history of fracture (no, yes), thyroid disease (no, yes), removing both ovaries (no, yes). Based on the likelihood ratio test, interaction analysis was used to test the differences between subgroups.

Several sensitivity analyses were conducted to evaluate the robustness of the findings. Firstly, considering the possible effect of disease on OP/ osteopenia, we further excluded individuals who had gout, liver disease, kidney disease, thyroid disease, removing both ovaries and a history of fracture. Secondly, we used unweighted data to perform sensitivity analysis.

Finally, mediation analysis was utilized to investigate whether BMI acts as a mediator in the association between the SUA and OP/ osteopenia. Bootstrap resampling was utilized, with 5000 repetitions, to rigorously examine the mediation effects.

Statistical analyses were performed with R statistical software (version 4.3.2). A p-value of < 0.05 (2-tailed) was considered statistically significant in all analyses.

## Results

A total of 12,581 participants aged ≥ 18 years were included (NHANES 2007–2010, 2013–2014, 2017–2018), and the weighted number of participants was 118,404,199, with a mean age of 51.0 years old, comprising 53.4% men and 46.6% women. Of these individuals, the prevalence of OP/osteopenia was 45.9% (5776/12,581). Based on the weighted analyses, participants with OP/osteopenia were more likely to have low serum uric acid levels, older, female, non-Hispanic White, BMI < 30, high HDL, low ALT, low AST, low albumin, low bilirubin, high alkaline phosphatase, high serum 25(OH.)D, high phosphorus, low eGFR, high cholesterol, less likely to have vigorous work activity, and comorbidities (i.e., hypertension, kidney disease, thyroid disease, history of fracture, removing both ovaries), and detailed baseline characteristics were listed in Table 1.

**Table 1:** Characteristics of the study population, according to NHANES 2007–2010, 2013–2014, 2017–2018 (*n* = 12,581)

Characteristic	Participants^a^, No, (%)		
	Normal (*n* = 6805)	OP or osteopenia (*n* = 5776)	*P* value^b^
Sex			< 0.001
Male	4325(64.2)	2388(40.1)	
Female	2480(35.8)	3388(59.9)	
Age mean (SE), y	44.04 (14.86)	55.58 (14.69)	< 0.001
Race/Hispanic origin			< 0.001
Mexican American	1325(10.4)	938(6.9)	
Other Hispanic	789(6.2)	633(5.0)	
Non-Hispanic White	2512(62.4)	2780(72.6)	
Non-Hispanic Black	1715(14.5)	747(6.4)	
Other Race - Including Multi-Racial	464(6.5)	678(9.1)	
BMI			< 0.001
< 30	3869(57.6)	4357(75.2)	
≥ 30	2936(42.4)	1419(24.8)	
Serum uric acid (umol/L)			< 0.001
Q1 (≤ 267.7)	1508(21.0)	1826(32.8)	
Q2 (267.8-321.2)	1598(22.9)	1518(26.3)	
Q3 (321.3-380.7)	1839(28.2)	1285(22.9)	
Q4 (≥ 380.8)	1860(27.9)	1147(18.0)	
HDL, mean (SE), mmol/L	1.29 (0.38)	1.46 (0.44)	< 0.001
ALT, mean (SE), U/L	27.43 (21.57)	23.64 (14.47)	< 0.001
AST, mean (SE), U/L	26.04 (17.16)	25.06 (13.61)	0.011
Albumin, mean (SE), g/L	42.95 (3.20)	42.36 (3.13)	< 0.001
Globulin, mean (SE), g/L	28.51 (4.20)	28.30 (4.39)	0.07
Bilirubin, mean (SE), umol/L	12.90 (5.58)	11.64 (5.25)	< 0.001
Alkaline phosphatase, mean (SE), U/L	67.37 (21.33)	71.45 (23.41)	< 0.001
Serum calcium, mean (SE), mmol/L	2.36 (0.09)	2.36 (0.09)	0.346
Serum 25(OH)D, mean (SE), nmol/L	66.09 (24.83)	72.97 (28.72)	< 0.001
Phosphorus, mean (SE), mmol/L	1.20 (0.19)	1.22 (0.18)	< 0.001
eGFR, mean (SE), ml/min*1.73m^2^	108.80 (57.77)	86.99 (64.59)	< 0.001
Total cholesterol, mean (SE), mmol/L	5.00 (1.05)	5.13 (1.08)	< 0.001
Diabetes			0.332
No	5632(86.5)	4616(85.7)	
Yes	1173(13.5)	1160(14.3)	
Hypertension			< 0.001
No	4495(69.1)	3284(61.6)	
Yes	2310(30.9)	2492(38.4)	
Vigorous work activity			< 0.001
No	5180(74.0)	4881(82.4)	
Yes	1625(26.0)	895(17.6)	
Self-reported disease			
Gout			0.243
No	6491(95.9)	5487(95.4)	
Yes	314(4.1)	289(4.6)	
Liver disease			0.14
No	6577(96.5)	5501(95.7)	
Yes	228(3.5)	275(4.3)	
Kidney disease			0.015
No	6657(98.4)	5589(97.6)	
Yes	148(1.6)	187(2.4)	
History of fracture			< 0.001
No	6218(89.5)	5024(85.5)	
Yes	587(10.5)	752(14.5)	
Thyroid disease			< 0.001
No	6316(92.3)	5006(86.0)	
Yes	489(7.7)	770(14.0)	
Removing both ovaries			< 0.001
No	6637(97.8)	5266(91.0)	
Yes	168(2.2)	510(9.0)	

Abbreviation: NHANES, National Health and Nutrition Examination Survey; OP, osteoporosis; BMI, body mass index, calculated as weight in kilograms divided by height in meters squared; HDL, high-density lipoprotein; ALT, alanine aminotransferase; AST, asparate aminotransferase

^a^ All estimates accounted for complex survey designs, and all percentages were weighted

^b^ P values were computed separately for each covariate and indicate statistically significant differences between the two groups if *P* < 0.05

The results of sample-weighted multivariate logistic regression analyses are presented in Table 2. With the SUA classification as the only covariate, Setting Q1 as the reference, the ORs applied by the unadjusted univariate logistic regression model was 0.74 (95% CI = 0.65–0.84) for Q2, 0.52 (95% CI = 0.44–0.61) for Q3, 0.41 (95% CI = 0.36–0.48) for Q4. After adjustment for potential confounders, compared with Q1, results, except for Q2, indicated higher SUA concentrations were related to a lower incidence of OP/osteopenia (Q3 [OR = 0.74, 95%CI = 0.60–0.93], Q4 [OR = 0.63, 95%CI = 0.51–0.77]).

**Table 2:** Association between serum uric acid and osteoporosis or osteopenia

Serum uric acid classification	Weighted Odds Ratio (95%CI)		
	Model 1^a^	Model 2^b^	Model 3^c^
Q1	Ref(1.000)	Ref(1.000)	Ref(1.000)
Q2	0.74(0.65–0.84)	0.80(0.70–0.92)	0.90(0.77–1.04)
Q3	0.52(0.44–0.61)	0.61(0.50–0.74)	0.74(0.60–0.93)
Q4	0.41(0.36–0.48)	0.47(0.39–0.57)	0.63(0.51–0.77)
*P* for trend	< 0.001	< 0.001	< 0.001

Model 1^a^: crude model

Model 2^b^: adjusted for baseline sex, age, race

Model 3^c^: adjusted for baseline sex, age, race, body mass index, high-density lipoprotein, alanine aminotransferase, asparate aminotransferase, albumin, bilirubin, alkaline phosphatase, serum calcium, serum 25(OH)D, phosphorus, eGFR, total cholesterol, diabetes, hypertension, vigorous work activity, self-reported disease: gout, liver disease, kidney disease, history of fracture, thyroid disease, removing both ovaries

**Fig. 2:**
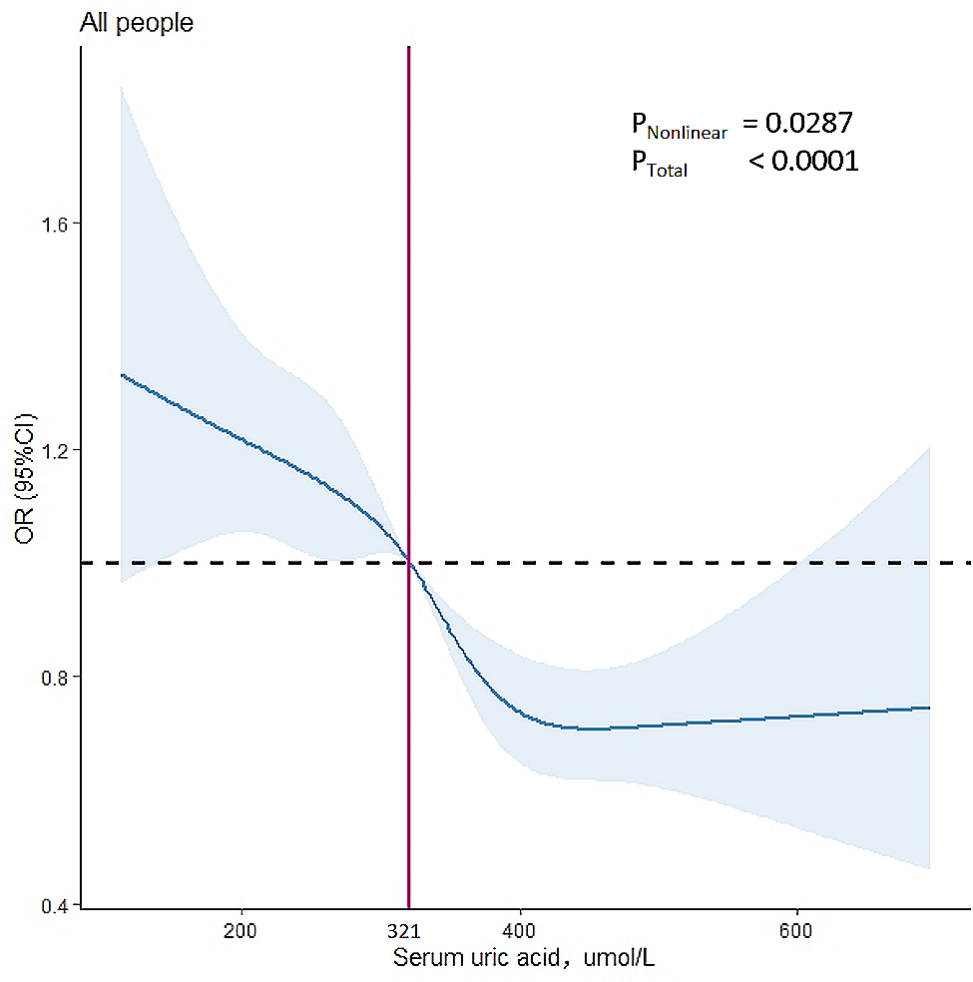
Association between SUA and osteoporosis/osteopenia in all 12,581 participants. A restricted cubic spline was modeled. Analysis was adjusted for sex, age, race, body mass index (BMI), high-density lipoprotein, alanine aminotransferase, asparate aminotransferase, albumin, bilirubin, alkaline phosphatase, serum calcium, serum 25(OH)D, phosphorus, eGFR, total cholesterol, diabetes, hypertension, vigorous work activity, self-reported disease: gout, liver disease, kidney disease, history of fracture, thyroid disease, and removing both ovaries. OR, odds ratio; CI, confidence interval; the analysis was weighted

A sample-weighted restricted cubic spline (RCS) model with optimal knots was used to estimate the dose-response relationship between SUA and the prevalence of OP/osteopenia in Fig. 2. We observed a U-shaped relationship between SUA and OP/osteopenia after adjusting for potential confounders (*P* < 0.0001, *P* for nonlinear = 0.0287). The risk of OP/osteopenia decreased slowly below an SUA of 321 µmol/L and decreased rapidly, which reached the lowest risk around 452 µmol/L, then slowly increased.

**Fig. 3:**
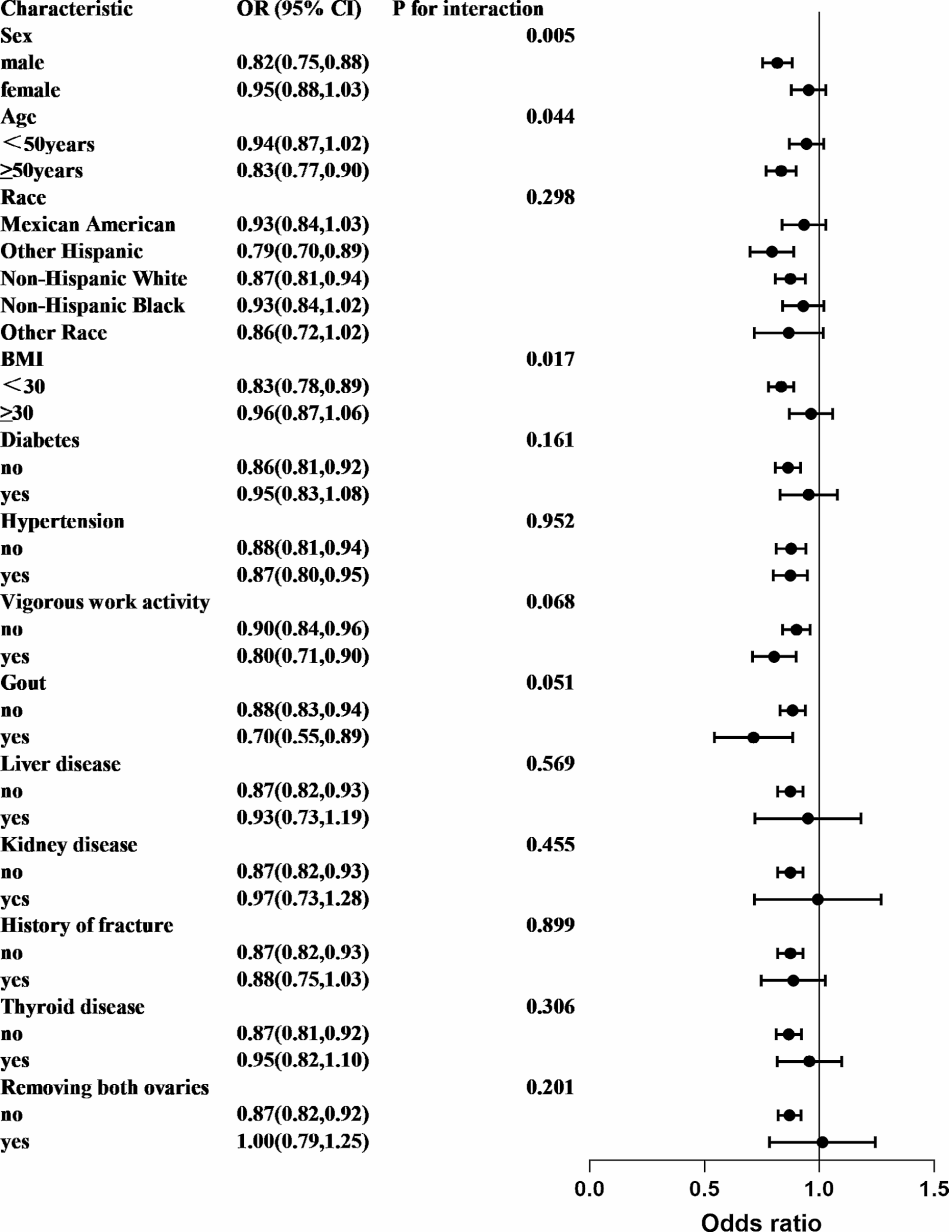
Forest plot for performance on the odds ratio of osteoporosis/osteopenia by serum uric acid in the subgroup. Each stratification was adjusted for sex, age, race, body mass index (BMI), high-density lipoprotein, alanine aminotransferase, asparate aminotransferase, albumin, bilirubin, alkaline phosphatase, serum calcium, serum 25(OH)D, phosphorus, eGFR, total cholesterol, diabetes, hypertension, vigorous work activity, self-reported disease: gout, liver disease, kidney disease, history of fracture, thyroid disease, and removing both ovaries except the stratification factor itself. Circles indicate odds ratios (ORs), with horizontal lines indicating 95% Cis, the analysis was weighted

**Fig. 4:**
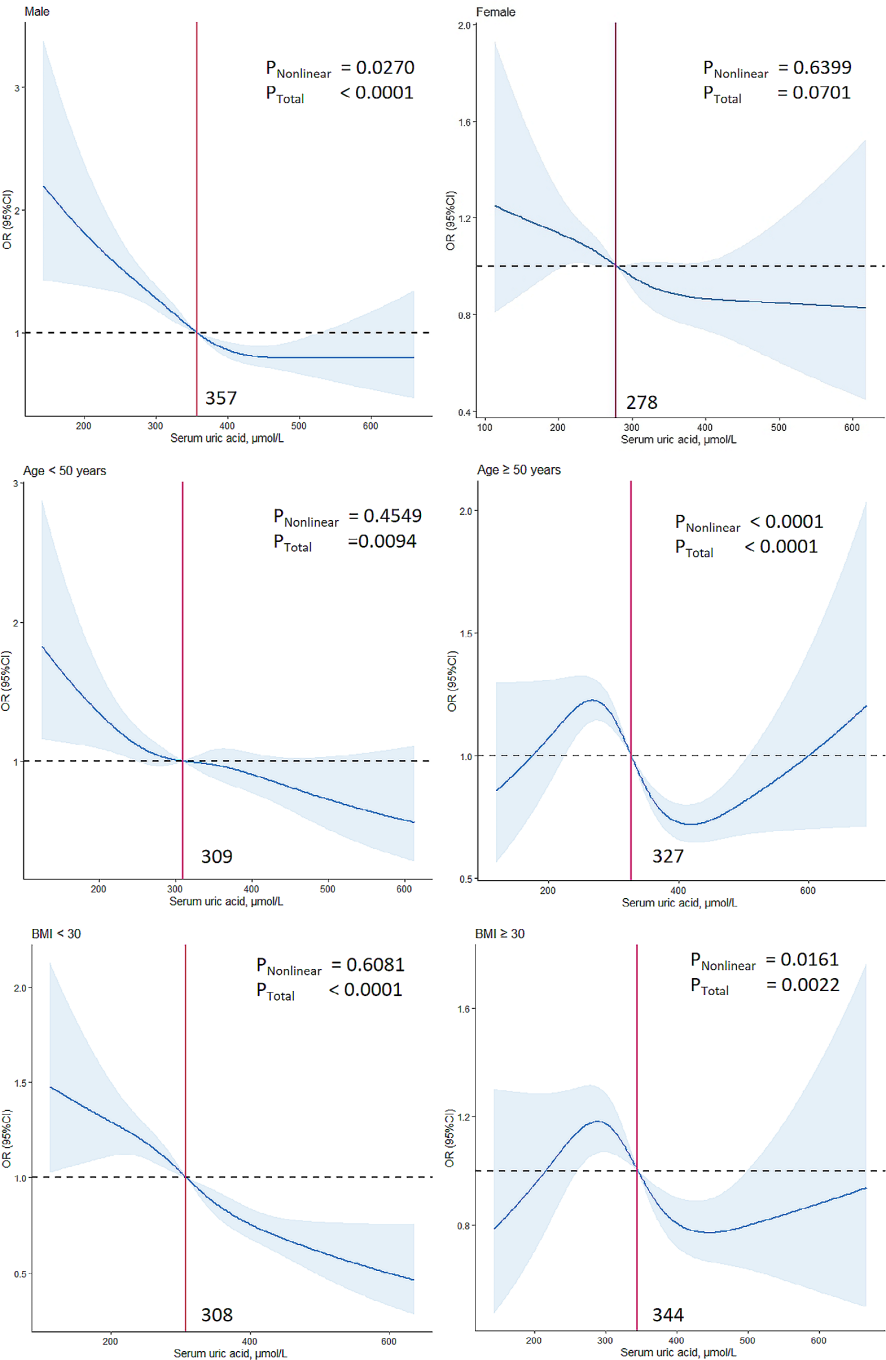
Association between SUA and osteoporosis/osteopenia in the different subgroups. A restricted cubic spline was modeled. Each stratification was adjusted for sex, age, race, body mass index (BMI), high-density lipoprotein, alanine aminotransferase, asparate aminotransferase, albumin, bilirubin, alkaline phosphatase, serum calcium, serum 25(OH)D, phosphorus, eGFR, total cholesterol, diabetes, hypertension, vigorous work activity, self-reported disease: gout, liver disease, kidney disease, history of fracture, thyroid disease, and removing both ovaries except the stratification factor itself. OR, odds ratio; CI, confidence interval, analyzes are weighted

The results of subgroup analyses are presented in Fig. 3. There were significant interactions in the association between SUA and OP/osteopenia for age subgroups (*P* for interaction = 0.044), sex subgroups (*P* for interaction = 0.005), and BMI subgroups (*P* for interaction = 0.017). Encouraged by the results, we further assessed whether there was a linear or nonlinear association between SUA and OP/osteopenia using multivariate-adjusted RCS based on the stratification of age, sex, and BMI (Fig. 4). In males, we found a L-shaped relationship between SUA and OP/osteopenia (*P* < 0.0001, *P* for nonlinear = 0.0270), with the increase of SUA, the ORs of OP/osteopenia presented a general trend of decreased. When the SUA levels were above 357 µmol/L, the ORs were significantly lower than 1.00, and the risk of OP/osteopenia decreased with the increase in SUA, with little evidence of correlation at SUA levels above 535 µmol/L. In people aged ≥ 50 years, the result showed that SUA and OP/osteopenia presented a N-shape nonlinear relationship (*P* < 0.0001, *P* for nonlinear < 0.0001), and when the SUA levels were above 327 µmol/L, the ORs were significantly lower than 1.00, the risk of OP/osteopenia decreased with the increase in SUA, which reached the lowest risk around 417 µmol/L, then increased. In obese people (BMI ≥ 30), an N-shaped nonlinear relationship was also observed (*P* = 0.0022, *P* for nonlinear = 0.0161). When the SUA levels were above 344 µmol/L, the ORs were significantly lower than 1.00, and the risk of OP/osteopenia decreased with the increase in SUA, which reached the lowest risk around 445 µmol/L, then increased. We estimated a linear inverse association between SUA and the risk of OP/osteopenia in people aged < 50 years (*P* = 0.0094, *P* for nonlinear = 0.4549) and with BMI < 30 (*P* < 0.0001, *P* for nonlinear = 0.6081), and the ORs were significantly lower than 1.00 at SUA levels above 309 µmol/L and 308 µmol/L, respectively. In females, the relationship between SUA and OP/osteopenia is complex, and we found no association after adjusting for variables (*P* = 0.0701, *P* for nonlinear = 0.6399), but excluding females removing both ovaries, there was a linear relationship between SUA and OP/osteopenia (*P* = 0.0288, *P* for nonlinear = 0.2892), and among females with both ovaries removed, we found no association (*P* = 0.3818, *P* for nonlinear = 0.9798) (Supplement 1). In addition, a linear relationship was also observed in males aged < 50 years, males with BMI < 30, and males with BMI ≥ 30, and a U-shaped association was found in males aged ≥ 50 years (Supplement 2).

The results of sensitivity analyses are summarized in Table 3. After excluding participants who had gout, liver disease, kidney disease, history of fracture, thyroid disease, and removing both ovaries, higher SUA levels were associated with OP/osteopenia. In the unweighted analysis, the results were generally robust.

**Table 3:** Sensitivity Analyses

Analysis	Unweighted participants/total participants, No.	Adjusted OR (95% CI)	*P* value
Excluding participants had some disease^a^			
SUA Q1	1193/2371	Ref(1.000)	< 0.001
SUA Q2	983/2222	0.91(0.77–1.09)	
SUA Q3	818/2219	0.72(0.57–0.91)	
SUA Q4	628/1970	0.63(0.49–0.80)	
Unweighted analyses^b^			
SUA Q1	1826/3334	Ref(1.000)	< 0.001
SUA Q2	1518/3116	0.94(0.84–1.06)	
SUA Q3	1285/3124	0.80(0.70–0.91)	
SUA Q4	1147/3007	0.73(0.64–0.84)	

Abbreviations: SUA, serum uric acid; OR, odds ratio

^a^ Included the possible effect of disease on bone health: gout, liver disease, kidney disease, thyroid disease, removing both ovaries, history of fracture. Adjusted for baseline sex, age, race, body mass index, high-density lipoprotein, alanine aminotransferase, asparate aminotransferase, albumin, bilirubin, alkaline phosphatase, serum calcium, serum 25(OH)D, phosphorus, eGFR, total cholesterol, diabetes, hypertension, vigorous work activity

^b^ Adjusted for baseline sex, age, race, body mass index, high-density lipoprotein, alanine aminotransferase, asparate aminotransferase, albumin, bilirubin, alkaline phosphatase, serum calcium, serum 25(OH)D, phosphorus, eGFR, total cholesterol, diabetes, hypertension, vigorous work activity, self-reported disease: gout, liver disease, kidney disease, history of fracture, thyroid disease, removing both ovaries

**Fig. 5:**
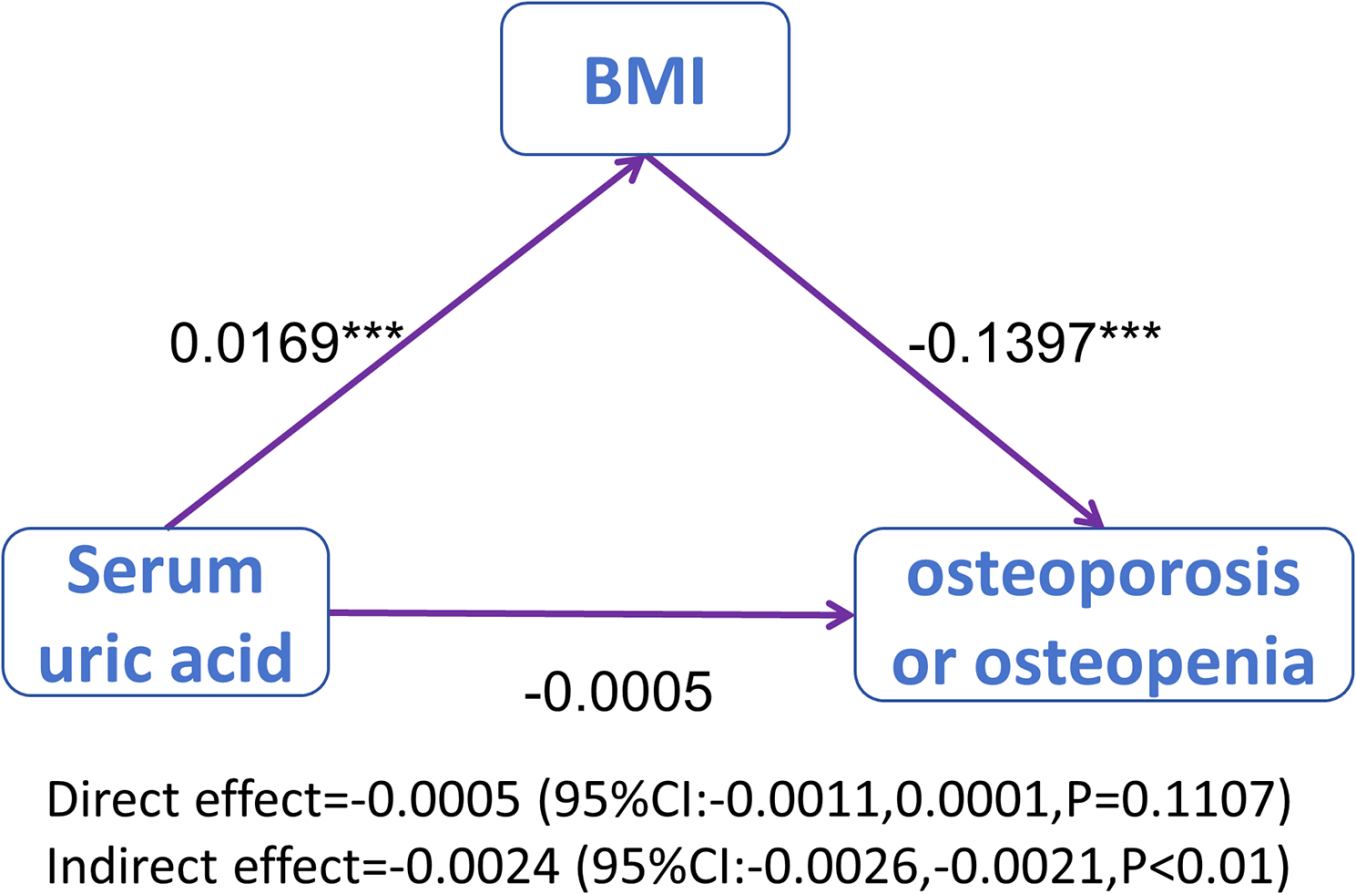
Mediation analysis. Sex, age, race, high-density lipoprotein, alanine aminotransferase, asparate aminotransferase, albumin, bilirubin, alkaline phosphatase, serum calcium, serum 25(OH)D, phosphorus, eGFR, total cholesterol, diabetes, hypertension, vigorous work activity, gout, liver disease, kidney disease, history of fracture, thyroid disease, and removing both ovaries were included as covariate variables, the analysis was unweighted, ***P < 0.001

Sex, age, race, high-density lipoprotein, alanine aminotransferase, asparate aminotransferase, albumin, bilirubin, alkaline phosphatase, serum calcium, serum 25(OH)D, phosphorus, eGFR, total cholesterol, diabetes, hypertension, vigorous work activity, gout, liver disease, kidney disease, history of fracture, thyroid disease, and removing both ovaries were included as covariates, we further evaluated whether BMI mediate the association between SUA and OP/osteopenia. As shown in Fig. 5, BMI fully mediated the association of SUA and OP/osteopenia, with a value of -0.0024(-0.0026–-0.0021).

## Discussion

In the present study, we observed a U-shaped association between SUA levels and OP/osteopenia in all populations, with either too low or too high SUA levels associated with increased risk. We further estimated the subgroups in which significant interactions were observed and found that the optimal range of SUA levels was 357–535 µmol/L in males, 327–417 µmol/L in people aged ≥ 50 years, above 309 µmol/L in people aged < 50 years, 344–445 µmol/L in people with BMI ≥ 30, and above 308 µmol/L in people with BMI < 30. The relationship between SUA and OP/osteopenia in the females may be influenced by estrogen and needs to be treated with caution. Furthermore, the possible fully mediating role of BMI in the association between SUA and OP/osteopenia was found.

Currently, there is a controversial relationship between SUA and bone health in the existing clinical studies. A meta-analysis suggested that higher SUA levels have a protective role in bone metabolism disorders [18]. Several studies reported a positive correlation between SUA and BMD in older people [19, 20]. A Rotterdam study (10.9-year follow-up) in 5074 women and men reported higher SUA levels were associated with higher femoral neck BMD and a lower risk of incident osteoporotic fracture [21]. A study by Kaushal et al. from a healthy Indian population measured BMD at five bone sites (lumbar spine (L1–L4), femoral neck (both right and left), and total femur (both right and left)) showed a significant positive association between UA and BMD at all bone sites [22]. Our results also showed that slightly higher SUA was associated with higher BMD and a lower risk of OP or osteopenia, consistent with the above results.

In contrast, a cross-sectional study using multivariate linear regression to analyze 6704 adult males from NHANES 1999–2006 demonstrated no association was found between SUA and lumbar spine BMD [11]. Zhao et al. [23] used Logistic regression to analyze the factors influencing osteoporosis in postmenopausal women with type 2 diabetes and showed that SUA was neither a protective nor a risk factor for osteoporosis. A multiple linear regression analysis on 328 postmenopausal women reported no significant correlation was observed between SUA and BMD [24]. The potential reasons for this discrepancy might be the differences in demographic characteristics and the use of statistical methods.

The study populations of some studies were women who developed estrogen deficiency in postmenopausal or ovarian removed. Estrogen is one of the key regulators of bone metabolism, and its deficiency predisposes a disease in BMD [25]. In our study, there was no relationship between SUA and OP/osteopenia in women, and further analysis found that a negative association was observed after exclusion women removing both ovaries, but not in women with both ovarian resection, suggesting that estrogen may have played a key role. At the same time, estrogen also has a certain effect on SUA by promoting the production of uric acid and inhibiting its excretion. Therefore, more studies are needed to examine the mediating role of estrogen in the relationships between SUA and bone health.

In addition, all the above results were based on linear or transformed linear (log-transformed) models to explore the relationship between SUA and BMD, which forced a linear association between SUA and BMD. However, in the real world, many elements in the human body stay within a range of levels that are neither too low nor too high with a usual pattern of U-shaped nonlinear manner. Those forced linear analyses may fail to capture the complex potential features, as evidenced by the extensive research discrepancies [26]. Our study further explored the dose-response relationship using the nonlinear RCS model, implying that there may be an optimal beneficial range of SUA for bone health.

Interestingly, a two-sample Mendelian randomized study from European descent and the UK biobank found no causal association between SUA and BMD by measuring single nucleotide polymorphism [10]. A similar conclusion was obtained by Dalbeth N et al. [27]. However, the study found that some of the urate transporters included within the genetic urate score (particularly ABCG229) were widely expressed [27]. Urate transporters might affect bone turnover and BMD by affecting the transport of other substrates that influence bone biology [27]. Furthermore, the method has limitations because the uric acid level is not only determined by genetic factors but also susceptible to eating habits, alcohol consumption or obesity status, etc. In addition to the multiple biological effects of genetic variation, these effects may independently affect BMD.

Notably, the results of intermediary analysis suggested that fully of the protective effects of SUA on OP/osteopenia could be realized through its effects on BMI. The positive correlation between BMI and SUA has been confirmed by many previous studies [28, 29]. Studies have shown that BMI positively correlates with BMD [30–32]. The prevalence of OP/osteopenia in obese patients was 25.6%, which was much lower than that in non-obese patients (46.9%) in our study. Obese people have increased body fat and lean mass, leading not only to passive loading but also to increased muscle strain, which has a beneficial effect on bone health [33]. These findings might have constituted a supporting element for the outcomes of our intermediary analysis.

The antioxidant effect of UA has been demonstrated by many studies [7, 34, 35], which inhibits osteoclast bone resorption and promotes bone formation by effectively scavenging free radicals in human plasma. But this benefit might be disturbed by the hydrophobic lipid layer of the cell membrane, and the oxidized lipids could convert uric acid into an oxidant with the help of copper [35–37]. Meanwhile, another mechanism proposed that UA produced intracellular free radicals during the degradation process, which further enhanced superoxide by interacting with NADPH oxidase [35]. This in-tracellular oxidative stress, together with UA-induced inflammatory cytokines, stimu-lates osteoclast bone resorption and inhibits osteoblastic bone formation leading to bone loss [38, 39]. Additionally, SUA may affect 1, 25D, and PTH levels, which can adversely affect bone health [40]. The imbalance between oxida-tive stress and antioxidant is an important cause of affecting bone remodeling [35]. Obesity is closely related to oxidative stress and antioxidants [41]. Therefore, BMI may play an important role in the association between SUA and bone health, and further mechanical studies are needed.

The present study has several limitations. First, this is a cross-sectional study, and the risk of associated unmeasured confounders was not included, so we cannot evaluate the causal relationships of the associations between SUA and OP/osteopenia, so the results need to be treated with caution. Second, only the BMD of the total femur and femoral neck was analyzed in our study, and this may lead to a slightly different conclusion from other sites. Third, though a large population in our study, this was only a national study, and further prospective large-sample studies from multiple countries and underlying mechanistic studies are warranted to determine the exact impact of the association between SUA and bone health.

## Conclusions

In conclusion, this study indicated a complicated relationship between SUA and OP/ osteopenia in different populations and a significant negative association between a specific range of SUA and OP/osteopenia. In addition, BMI may play an important role in the association between SUA and bone health.

### Electronic supplementary material

Below is the link to the electronic supplementary material.

Supplementary Material 1: Association between SUA and osteoporosis/osteopenia in females (excluding females removing both ovaries and females with both ovaries removed). A restricted cubic spline was modeled. Analysis was adjusted for age, race, body mass index (BMI), high-density lipoprotein, alanine aminotransferase, asparate aminotransferase, albumin, bilirubin, alkaline phosphatase, serum calcium, serum 25(OH)D, phosphorus, eGFR, total cholesterol, diabetes, hypertension, vigorous work activity, self-reported disease: gout, liver disease, kidney disease, history of fracture, and thyroid disease. OR, odds ratio; CI, confidence interval, the analysis excluding females who have had both ovaries removed was weighted, the analysis of females with both ovaries removed was unweighted.

Supplementary Material 2: Association between SUA and osteoporosis/osteopenia in the different characteristics of males. A restricted cubic spline was modeled. Each stratification was adjusted for age, race, body mass index (BMI), high-density lipoprotein, alanine aminotransferase, asparate aminotransferase, albumin, bilirubin, alkaline phosphatase, serum calcium, serum 25(OH)D, phosphorus, eGFR, total cholesterol, diabetes, hypertension, vigorous work activity, self-reported disease: gout, liver disease, kidney disease, history of fracture, and thyroid disease except the stratification factor itself. OR, odds ratio; CI, confidence interval, the analysis was weighted.

## Data Availability

Availability of data and materials: Data described in the manuscript, code book, and analytic code will be made publicly and freely available without restriction at https://www.cdc.gov/nchs/nhanes/index.htm.

## Abbreviations

NHANES: National Health and Nutrition Examination Survey
SUA: Serum uric acid
BMD: Bone mineral density
OR: Odds ratio
CI: Confidence intervals
