# Supplementary figures and images for: BMI mediates the association of serum uric acid with bone health: a cross-sectional study of the National Health and Nutrition Examination Survey (NHANES)
Source: BMC Musculoskelet Disord. 2024 Jun 19;25:482. doi: 10.1186/s12891-024-07595-8 (PMC11186245; doi:10.1186/s12891-024-07595-8)

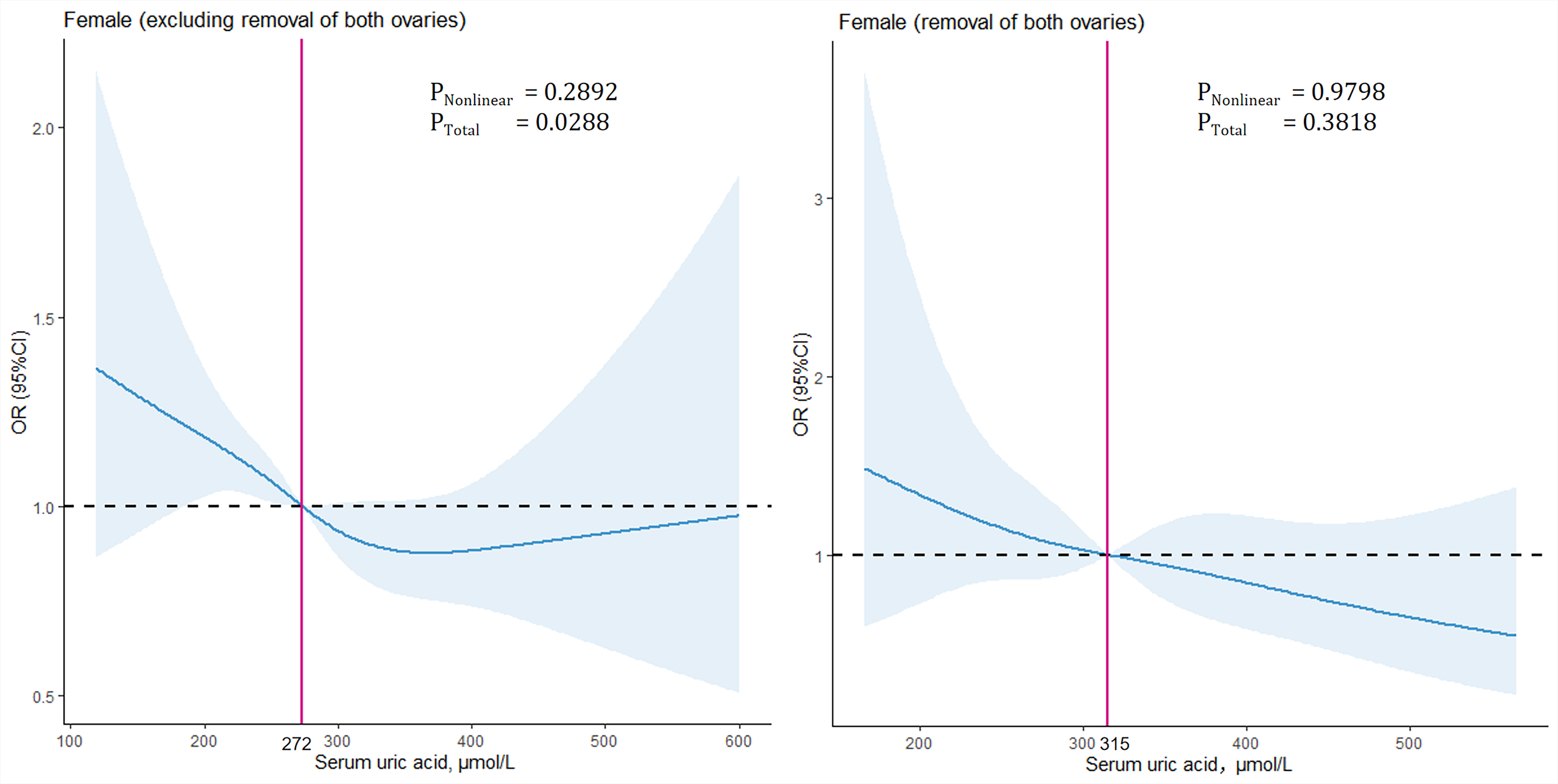

Supplement: Supplementary file 1 — Supplementary Material 1: Association between SUA and osteoporosis/osteopenia in females (excluding females removing both ovaries and females with both ovaries removed). A restricted cubic spline was modeled. Analysis was adjusted for age, race, body mass index (BMI), high-density lipoprotein, alanine aminotransferase, asparate aminotransferase, albumin, bilirubin, alkaline phosphatase, serum calcium, serum 25(OH)D, phosphorus, eGFR, total cholesterol, diabetes, hypertension, vigorous work activity, self-reported disease: gout, liver disease, kidney disease, history of fracture, and thyroid disease. OR, odds ratio; CI, confidence interval, the analysis excluding females who have had both ovaries removed was weighted, the analysis of females with both ovaries removed was unweighted. [file 12891_2024_7595_MOESM1_ESM.tif]

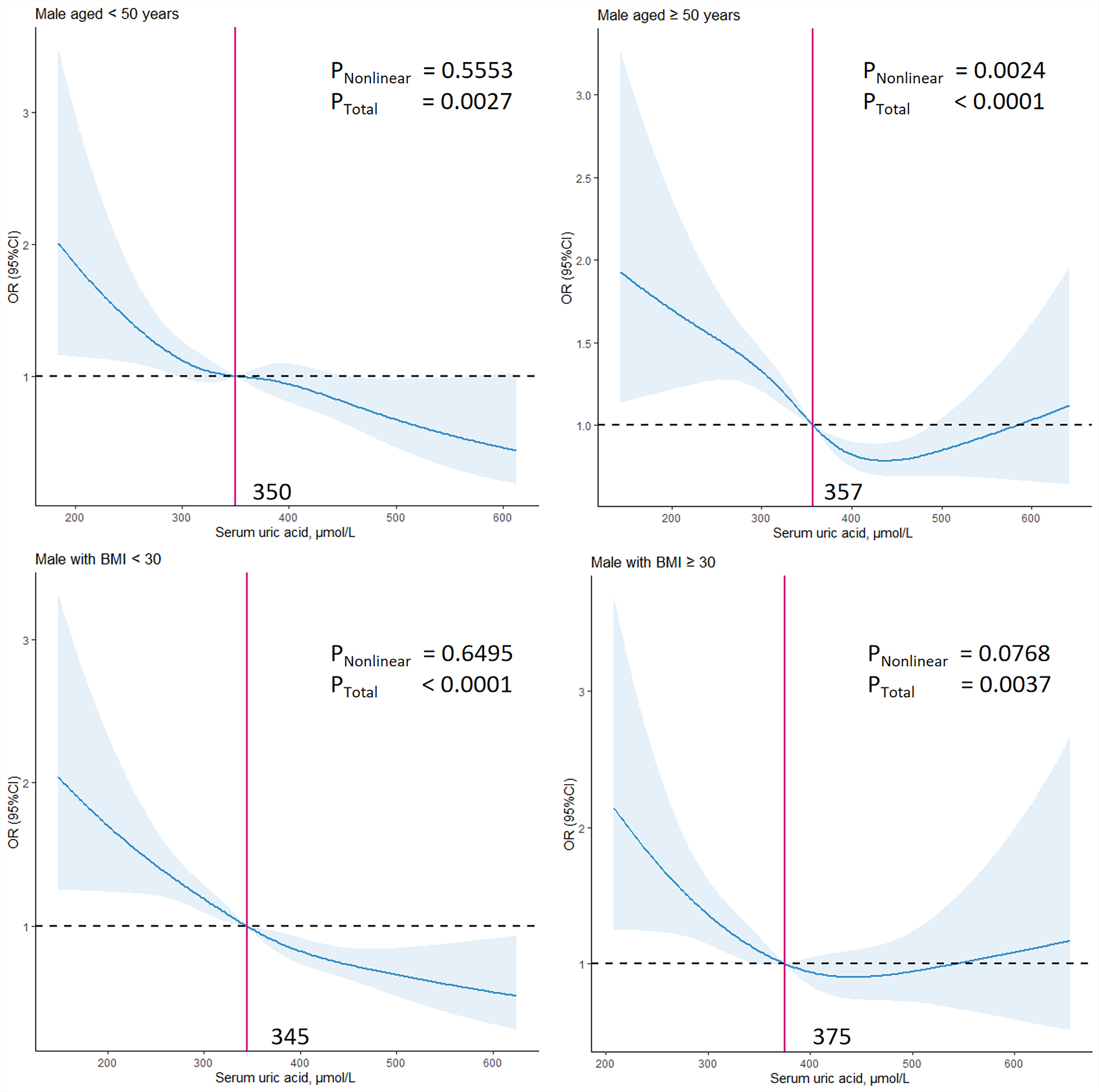

Supplement: Supplementary file 2 — Supplementary Material 2: Association between SUA and osteoporosis/osteopenia in the different characteristics of males. A restricted cubic spline was modeled. Each stratification was adjusted for age, race, body mass index (BMI), high-density lipoprotein, alanine aminotransferase, asparate aminotransferase, albumin, bilirubin, alkaline phosphatase, serum calcium, serum 25(OH)D, phosphorus, eGFR, total cholesterol, diabetes, hypertension, vigorous work activity, self-reported disease: gout, liver disease, kidney disease, history of fracture, and thyroid disease except the stratification factor itself. OR, odds ratio; CI, confidence interval, the analysis was weighted. [file 12891_2024_7595_MOESM2_ESM.tif]
